# Https://cpaper.ctimeetingtech.com/epa21/submission/edit

**DOI:** 10.1192/j.eurpsy.2021.2188

**Published:** 2021-08-13

**Authors:** D. Alonzo

**Affiliations:** Suicide Prevention Research Program, Fordham University, West Harrison, United States of America

**Keywords:** Integrated Motivational Volitional Model, Volitional factors, suicide attempters, Suicide

## Abstract

**Introduction:**

During the last 15 years, an ideation-to-action framework has been proposed that has prompted the development of several models that account for the transition from ideator to attempter. Previous research on one such model, the Integrated Motivational Volitional Model of Suicide (IMV), suggests it accurately distinguishes between suicide ideators and attempters. However, no study has examined the utility of the model with a psychiatric sample of depressed suicide ideators and attempters.

**Objectives:**

To address this gap in previous research, this study examines the ability of the IMV to distinguish between depressed adult suicide ideators with and without a history of suicide attempt presenting to the emergency department.

**Methods:**

After providing informed consent and with the approval of the appropriate institutional review board, 68 adults presenting to the Emergency Department were recruited to participate in the study. Ideators and attempers were compared on sociodemographics, severity of depression/hopelessness/current suicide ideation, and volitional factors including, access, planning, exposure to family suicide, impulsivity, pain tolerance, fearlessness about death, and mental imagery of death. Group differences were evaluated using chi-square and t-tests. Multivariate group analyses were performed using logistic regression.

**Results:**

Of the regression analysis indicate that fearfulness about death significantly predicts suicide attempt history (OR=10.560, p=.05). Ethnicity was also found to significantly predict suicide attempt history (OR=0.67, p=.006). No other sociodemographic variables or volitional moderators were significant.

**Conclusions:**

Results of this study contribute to improving accuracy in this area and suggest that fearlessness about death should be routinely included in comprehensive suicide risk assessments.

**Disclosure:**

No significant relationships.

